# Supplementary material for: Research on stroke patients’ perception of recurrence risk: A scoping review protocol
Source: PLoS One. 2024 Dec 5;19(12):e0312189. doi: 10.1371/journal.pone.0312189 (PMC11620677; doi:10.1371/journal.pone.0312189)
Supplement: S1 Checklist — (DOCX) [file pone.0312189.s001.docx]

**PRISMA-P (Preferred Reporting Items for Systematic review and Meta-Analysis Protocols) 2015 checklist: recommended items to address in a systematic review protocol***

| Section and topic | Item No | Checklist item |
| --- | --- | --- |
| ADMINISTRATIVE INFORMATION | | |
| Title: |  |  |
| Identification | 1a | This document is the protocol for a systematic review on stroke patients' perception of recurrence risk. |
| Update | 1b | N/A (This is not an update of a previous systematic review). |
| Registration | 2 | This protocol has been registered on Open Science Framework (OSF). Relevant materials and potential following updates are available at https://osf.io/7kq5t. |
| Authors: |  |  |
| Contact | 3a | Shanshan Zhu, School of Nursing, Xinxiang Medical University, Email: Zhushanshanvip@163.com  Xueting Sun, School of Nursing, Xinxiang Medical University, Email: 3054292813@qq.com  Xin Guo, School of Nursing, Xinxiang Medical University, Email: 907827461@qq.com  Meiqi Xu, Nursing Department, Fifth Clinical College of Xinxiang Medical University, Email: 1149170562@qq.com  Dingding Li, School of Nursing, Xinxiang Medical University, Email: 1276979684@qq.com  Shuaiyou Wang, School of Nursing, Xinxiang Medical University, Email: 1584182565@qq.com  Yage Shi, School of Nursing, Xinxiang Medical University, Email: 2791770549@qq.com  Chenjun Liu, School of Nursing, Xinxiang Medical University, Email: 1362043939@qq.com  Hongru Wang, Nursing Department, Fifth Clinical College of Xinxiang Medical University, Email: hlb333333@163.com  Huimin Zhang, School of Nursing, Xinxiang Medical University, Email: 35290915@qq.com  Corresponding Author:  Huimin Zhang, School of Nursing, Xinxiang Medical University, 601 East Jinsui Avenue, Xinxiang 453003, Henan, China |
| Contributions | 3b | **Conceptualization:** Shanshan Zhu, Xin Guo, Dingding Li.  **Funding acquisition:** Huimin Zhang, Hongru Wang.  **Methodology:** Xueting Sun, Shuaiyou Wang, Yage Shi.  **Project administration:** Huimin Zhang, Hongru Wang.  **Supervision:** Huimin Zhang, Hongru Wang.  **Writing – original draft:** Chenjun Liu, Shanshan Zhu.  **Writing – review & editing:** Huimin Zhang, Hongru Wang, Dingding Li. |
| Amendments | 4 | This is a new protocol and not an amendment of a previously completed or published protocol. Any important amendments to this protocol will be documented and dated. These amendments will include a description of the change and the rationale for the change, and they will be archived in the study files. |
| Support: |  |  |
| Sources | 5a | IThis work is supported by the [Key Scientific Research Project of Higher Education Institutions of Henan Province] (grant number: 23B320002), [Graduate Education Reform Project of Henan Province] (grant number: 2023SJGLX236Y), and [Postgraduate Education Reform and Quality Improvement Project of Henan Province] (grant number: YJS2024JC27). |
| Sponsor | 5b | The sponsors for this review are the Key Scientific Research Project of Higher Education Institutions of Henan Province, the Graduate Education Reform Project of Henan Province, and the Postgraduate Education Reform and Quality Improvement Project of Henan Province. |
| Role of sponsor or funder | 5c | The funders and sponsors had no role in the design, execution, interpretation, or writing of the study. Their support was limited to providing financial assistance for the research. |
| INTRODUCTION | | |
| Rationale | 6 | Stroke is a major global cause of death and disability with a high recurrence rate that significantly affects the physical, psychological, and economic well-being of patients. Despite the importance of health risk perception in preventive measures, most stroke patients struggle to accurately assess their risk of recurrence. Current research on stroke recurrence risk perception is still exploratory, with a lack of systematic understanding of the influencing factors. |
| Objectives | 7 | The primary objective of this systematic review is to assess the perception of recurrence risk among stroke patients. Specifically, this review will address the following questions:  - Participants: What are the characteristics of stroke patients included in the studies?  - Interventions: What methods are used to assess patients' perception of recurrence risk?  - Comparators: How do different subgroups (e.g., based on age, gender, stroke severity) perceive recurrence risk differently?  - Outcomes: What are the reported levels of perceived recurrence risk, and what factors are associated with higher or lower perceived risk? |
| METHODS | | |
| Eligibility criteria | 8 | **Inclusion**  The inclusion criteria were based on the study population, concept, context, and type of evidence source.  **1.Population:** Stroke patients aged 18 years and older, including those with ischemic and hemorrhagic strokes, without restrictions on gender, ethnicity, geographical location, or time since diagnosis.  **2.Concept**: Studies that explore stroke patients' perception of recurrence risk, including risk awareness (understanding and knowledge of the likelihood of recurrence), risk attitudes (emotional responses towards the possibility of recurrence), risk management (actions and behaviors to manage and mitigate recurrence risk), and influencing factors (demographic, psychological, social, and clinical factors).  **3.Context:** Studies conducted in community health centers, hospitals, and home settings.  **4.Type of Evidence Source:** Available full-text publications and research papers, irrespective of research methodology or design, such as quantitative studies (interventional, cross-sectional, and longitudinal), qualitative studies, or mixed-methods research.  Publications in Chinese and English are included.  **Exclusion**  1. Documents that do not adequately address the research questions (e.g., guidelines, reviews, research proposals, and government documents).  2. Articles that are incomplete or where the full text is not accessible. |
| Information sources | 9 | Searches will be conducted across seven English-language databases (PubMed, CINAHL, Web of Science, Embase, Cochrane Library, PsycInfo, and MEDLINE) and four Chinese-language databases (CNKI, Wanfang, VIP, and China Biomedical Database). The search period will be from the inception of each database to December 2024. Additionally, reference lists of relevant studies and gray literature will be searched to achieve comprehensive retrieval. Before the final analysis, we will rerun the search to determine whether any newly relevant studies were included. |
| Search strategy | 10 | The search strategy was developed using MeSH terms and keyword combinations. An example of the PubMed search strategy is provided below:  #1 stroke[MeSH Terms]  #2 ((((stroke[Title/Abstract]) OR (strokes[Title/Abstract])) OR (stroke patient[Title/Abstract])) OR (poststroke[Title/Abstract])) OR (stroke survivor[Title/Abstract])  #3 #1 OR #2  #4 recurrence[MeSH Terms]  #5 ((recurrence[Title/Abstract]) OR (recurrences[Title/Abstract])) OR (relapse[Title/Abstract])  #6 #4 OR #5  #7 risk perception[MeSH Terms]  #8 (((risk perception[Title/Abstract]) OR (perceived risk[Title/Abstract])) OR (Perceived susceptibility[Title/Abstract])) OR (risk awareness[Title/Abstract])  #9 #7 OR #8  #10 #3 AND #6 AND #9 |
| Study records: |  |  |
| Data management | 11a | All retrieved literature will be managed using EndNote X9 software, and duplicates will be removed. Two researchers (SZ and XS) will independently screen titles, abstracts, and full texts to exclude irrelevant studies and ensure the inclusion of studies that meet the inclusion criteria. The entire literature retrieval and screening process will be detailed in the final scoping review and presented in a PRISMA flowchart. |
| Selection process | 11b | Two researchers (SZ and XS) will independently screen titles, abstracts, and full texts to exclude irrelevant studies and ensure the inclusion of studies that meet the inclusion criteria. In case of disagreements, a third researcher (HZ) will be consulted to reach a decision through discussion. |
| Data collection process | 11c | Data will be extracted using a standardized form developed in Excel. The form is designed to capture relevant information comprehensively and adequately. Data extraction will include items such as study author, year of publication, country/region, study population/sample size, research method/design, research themes, general condition of patients, data analysis methods, assessment tools, level of recurrence risk perception, and influencing factors. Data will be independently extracted by two authors, with discrepancies resolved through discussion among the review team. |
| Data items | 12 | Data items will include:  - Study author  - Year of publication  - Country/region  - Study population/sample size  - Research method/design  - Research themes  - General condition of patients  - Data analysis methods  - Assessment tools  - Level of recurrence risk perception  - Influencing factors |
| Outcomes and prioritization | 13 | Primary outcomes:  - Level of perceived recurrence risk  - Factors influencing recurrence risk perception  Additional outcomes:  - Methods used to assess patients' perception of recurrence risk  - Impact of perceived recurrence risk on patients' health behaviors and lifestyle |
| Risk of bias in individual studies | 14 | Given the exploratory nature of this scoping review, we will not perform a critical appraisal of individual evidence sources nor will we assess the quality of evidence in the included records. However, a section on the study limitations will be included to detail the shortcomings of this review. |
| Data synthesis | 15a | Data will be synthesized narratively and, where appropriate, using quantitative methods. Both qualitative and quantitative methods will be used for analysis, synthesis, and presentation. |
|  | 15b | Meta-analyses will not be conducted due to the scoping nature of this review. Instead, we will use descriptive statistics and narrative synthesis to summarize the findings. |
|  | 15c | Additional analyses, such as subgroup analyses, will be conducted if sufficient data are available. |
|  | 15d | A qualitative synthesis will be conducted to provide a comprehensive overview of the findings. |
| Meta-bias(es) | 16 | Publication bias will not be assessed due to the exploratory and descriptive nature of the scoping review. |
| Confidence in cumulative evidence | 17 | The strength of evidence will not be formally assessed in this scoping review due to its exploratory nature. However, we will discuss the limitations and strengths of the included studies in the final review. |

*** It is strongly recommended that this checklist be read in conjunction with the PRISMA-P Explanation and Elaboration (cite when available) for important clarification on the items. Amendments to a review protocol should be tracked and dated. The copyright for PRISMA-P (including checklist) is held by the PRISMA-P Group and is distributed under a Creative Commons Attribution Licence 4.0.**

*From: Shamseer L, Moher D, Clarke M, Ghersi D, Liberati A, Petticrew M, Shekelle P, Stewart L, PRISMA-P Group. Preferred reporting items for systematic review and meta-analysis protocols (PRISMA-P) 2015: elaboration and explanation. BMJ. 2015 Jan 2;349(jan02 1):g7647.*
