# Supplementary material for: Research on stroke patients’ perception of recurrence risk: A scoping review protocol
Source: PLoS One. 2024 Dec 5;19(12):e0312189. doi: 10.1371/journal.pone.0312189 (PMC11620677; doi:10.1371/journal.pone.0312189)
Supplement: Attachment — Submitted filename: Response to Reviewers.docx [file pone.0312189.s002.docx]

**Response to Reviewers**

Dear Dr. Apurva Kumar Pandya and Reviewers,

We would like to thank you for the thoughtful and constructive feedback on our manuscript entitled “Research on Stroke Patients' Perception of Recurrence Risk: A Scoping Review Protocol” (PONE-D-24-20180). We have carefully considered the comments from the academic editor and reviewer(s) and have revised the manuscript accordingly. Below, we provide a detailed response to each comment and explain the changes we have made in the revised version. All changes are highlighted in the manuscript using "Track Changes."

**Reviewer #1 Comments:**

**Comment 1:** The Protocol is explained in sufficient detail. However, in the section of data collection tool, there are certain typographical errors which need to be corrected.

**Response:** Thank you for pointing this out. We have carefully reviewed the entire manuscript and corrected all typographical errors, particularly in the section related to the data collection tool. These corrections are highlighted in the revised manuscript (see page 9, line 187 for the specific changes).

**Comment 2:** At places, the tenses of the sentences are changing too frequently which can be rechecked.

Response: We appreciate your suggestion regarding the consistency of tenses. We have thoroughly reviewed the manuscript and adjusted the tenses to maintain consistency throughout the text. The changes have been implemented and are visible in the revised manuscript.

**Comment 3:** The methodology of how the protocol will help identify the influencing factors is not clear and hence can be described in more detail.

Response: Thank you for this valuable feedback. We have added further clarification in the methods section to explain how the protocol will help identify influencing factors related to stroke patients' perception of recurrence risk. We have expanded on the description of the analysis framework and provided more specific details on the steps we will follow (see page 8, line 178-185 for the updated text).

**Editor's Additional Requirements:**

**Comment 4 (Financial Disclosure):** Please state what role the funders took in the study. If the funders had no role, please state: "The funders had no role in study design, data collection and analysis, decision to publish, or preparation of the manuscript."

Response: We confirm that the funders had no role in study design, data collection and analysis, decision to publish, or preparation of the manuscript. We have included the following statement in the cover letter and updated the financial disclosure accordingly: "The funders had no role in study design, data collection and analysis, decision to publish, or preparation of the manuscript."

**Comment 5 (Data Availability):** We strongly recommend all authors decide on a data sharing plan before acceptance. Please revise your data availability statement if necessary.

**Response:** We agree with the recommendation and have outlined a clear data sharing plan. Upon acceptance, all relevant data will be made available through a public repository, in accordance with PLOS ONE's open data policy.

**Comment 6 (Supporting Information):** Please include captions for your Supporting Information files at the end of your manuscript, and update any in-text citations to match accordingly.

**Response:** We have reviewed and updated the Supporting Information section to include the appropriate captions.

**Comment 7 (References):** Please review your reference list to ensure that it is complete and correct. If you have cited papers that have been retracted, please include the rationale for doing so or replace them with relevant current references.

**Response:** We have thoroughly reviewed our reference list and confirmed that all references are current and accurate. No retracted papers have been cited. If future revisions are necessary, we will update the reference list accordingly.

We hope that these revisions have addressed all concerns raised by the reviewer(s) and editor. Please let us know if further clarifications or changes are needed. We look forward to your feedback and appreciate your continued consideration of our manuscript.

Sincerely,

Huimin Zhang

Corresponding Author on behalf of all authors
